# Tick-Borne Bacteria and Protozoa in *Ixodes ricinus* Ticks Collected from Fallow Deer (*Dama dama*) in a Central Italian Protected Area

**DOI:** 10.3390/biology15080596

**Published:** 2026-04-09

**Authors:** Valentina Virginia Ebani, Fabrizio Bertelloni, Paolo Bongi, Chiara Trebino, Fabio Macchioni, Marco Del Frate, Marco Apollonio, Francesca Mancianti

**Affiliations:** 1 Department of Veterinary Science, University of Pisa, Viale delle Piagge 2, 56124 Pisa, Italy; fabrizio.bertelloni@unipi.it (F.B.); c.trebino@studenti.unipi.it (C.T.); fabio.macchioni@unipi.it (F.M.); francesca.mancianti@unipi.it (F.M.); 2 Centre for Climate Change Impact, University of Pisa, Via del Borghetto 80, 56124 Pisa, Italy; 3 Department of Veterinary Medicine, University of Sassari, 07100 Sassari, Italy; bongip73@yahoo.it (P.B.); delfratemarco@gmail.com (M.D.F.); marcoapo@uniss.it (M.A.)

**Keywords:** tick-borne pathogens, *Ixodes ricinus*, *Dama dama*, *Anaplasma phagocytophilum*, *Borrelia* sp., *Theileria* sp., zoonosis

## Abstract

In order to assess the spreading of tick-borne bacteria and protozoa in a protected area largely frequented by people and in which numerous domestic and wild animals live, molecular analyses were carried out in ticks collected from fallow deer (*Dama dama*) to detect *Anaplasma phagocytophilum*, *Borrelia* sp., *Coxiella burnetii*, *Francisella tularensis*, *Hepatozoon* sp., and piroplasms. A total of 148 tick pools, for a total of 475 ticks collected from fallow deer and identified as female adult *Ixodes ricinus*, were submitted to DNA extraction and different PCR assays. One hundred and two (68.92%) pools were positive for one or more pathogens: three (2.02%) for *C. burnetii*, 21 (14.19%) for *Borrelia* sp., 35 (23.64%) for piroplasms, and 87 (58.78%) for *A. phagocytophilum.* All tick pools were negative for *F. tularensis* and *Hepatozoon* sp. Sixty-seven (45.27%) pools were positive for only one investigated pathogen, whereas in 35 (23.64%) pools DNA of two or more pathogens were found. Sequencing analyses identified 28 piroplasm amplicons as *Theileria* sp. OT3 and seven amplicons as possible *Theileria cervi*. Sequencing of the 21 *Borrelia* amplicons identified six samples as *B. miyamotoi* and eight as *B. lusitaniae*, whereas seven amplicons had 100% homology with a *Borrelia* sp. found in France and 99.37% with a *B. theileri* strain detected in Zambia. Monitoring tick-borne pathogens in ticks is pivotal to assess the spread of these microorganisms, the evolution of their epidemiology, and the risk of infections for animals and humans.

## Simple Summary

Ticks are the most important hematophagous vectors of pathogens, most of which are zoonotic agents. The transmission of bacteria such as *Borrelia* sp., *Coxiella burnetii* and *Francisella tularensis*, which cause severe diseases in humans and animals, is often associated with tick bites. Other tick-borne pathogens, such as *Anaplasma phagocytophilum*, *Hepatozoon* sp., and piroplasms, usually cause diseases in domestic and wild animals. Tick-borne diseases are emerging due to the geographical expansion of their tick vectors and represent important threats for the health of animals, mainly pets, and humans. Monitoring tick-borne pathogens in tick populations is a pivotal tool for assessing the spread of these microorganisms, the evolution of their epidemiology, and the risk of infections for animals and humans. Molecular analyses on ticks collected from wild deer are important to obtain this information.

## 1. Introduction

Ticks are the most important hematophagous vectors of pathogens, most of which are zoonotic agents. Tick-borne diseases are emerging due to the geographical expansion of their tick vectors and represent important threats for the health of domestic and wild animals, and humans.

*Ixodes ricinus* ticks are highly competent vectors for a variety of pathogens, such as viruses, bacteria and protozoa, in humans and animals [1]. *Ixodes ricinus* can infest more than 300 different vertebrate species, including birds, lizards, small rodents, hares, hedgehogs, wild boars, carnivores, and ruminants [1]. Its life cycle involves larvae feeding predominantly on small mammals or birds, nymphs feeding on small and large mammals, and adults preferring larger mammals [1]. *Ixodes ricinus* is widespread in Europe, including Italy [2], mainly present in deciduous and mixed forests, although some studies found these ticks in green peri-urban and urban areas [3].

Tick abundance, in addition to being influenced by environmental factors such as climate, habitat characteristics, and trophic resource availability, is also associated with the abundance of their vertebrate hosts, particularly wild ungulate species. In North America, increases in deer abundance and distribution have strongly influenced the emergence and spread of the black-legged tick (*Ixodes scapularis*) and the lone star tick (*Amblyomma americanum*), as demonstrated in several studies [4,5,6]. However, the relationship between deer density and tick abundance is not linear. Indeed, Elias et al. [7] showed that beyond a certain threshold, further increases in deer density do not result in substantial increases in tick abundance, including the density of infected nymphs—an epidemiologically important metric used to estimate environmental risk for Lyme disease. On the other hand, the optimal deer population density required to reduce the incidence of Lyme disease in humans has not yet been clearly established. A density of approximately 3 deer/Km^2^ has been associated with a reduction in the zoonotic transmission of Lyme disease to humans [8]. Computer simulations have further suggested that maintaining deer densities at around 7.5 individuals/Km^2^ could result in a 40% reduction in the density of infected nymphs, whereas near-complete elimination of deer would be necessary to achieve a 90% reduction [9].

Bacteria such as *Borrelia burgdorferi* sensu lato (s.l.) and other *Borrelia* species, *Coxiella burnetii*, *Francisella tularensis*, and *Anaplasma phagocytophilum* and, among protozoa, a few *Babesia* species, are transmitted by ticks causing diseases in wild and domestic animals and often inducing severe zoonotic clinical forms [10,11]. Protozoa of the genus *Theileria*, which belongs to the order Piroplasmida along with the genus *Babesia*, and *Hepatozoon* sp., are other pathogens transmitted by ticks [10,11,12].

Monitoring tick-borne pathogens (TBP) in tick populations is a pivotal tool to assess the spreading of these microorganisms, evolution of their epidemiology, and risk of infections for animals and humans. At this purpose, the aim of the present investigation was to determine the occurrence of tick-borne protozoa and zoonotic bacteria in ticks collected from high-density fallow deer (*Dama dama*) population living in a protected area located in Central Italy, where wild animals are abundant and people spend time for recreational purposes. In detail, PCR assays were used to detect DNA of *A. phagocytophilum*, *Borrelia* sp., *C. burnetii*, *F. tularensis*, *Hepatozoon* sp., and piroplasms in ticks.

## 2. Materials and Methods

### 2.1. Sampling Area

**Figure 1:**
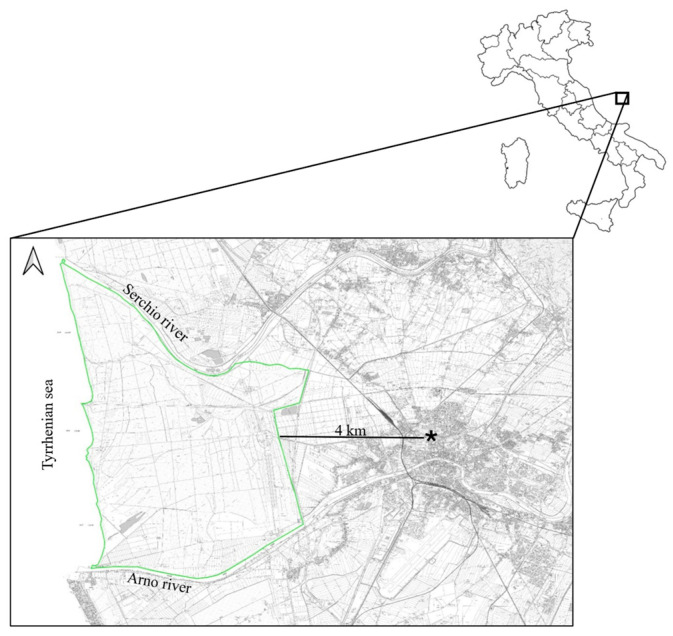
Map of the study area. The boundary of the study area is represented by a light green line, while the asterisk (*) indicates the Leaning Tower (from [14]).

All ticks analyzed were collected from wild fallow deer (*D. dama*) living in the San Rossore Estate, within the Migliarino–San Rossore–Massaciuccoli Regional Park (Tuscany, Central Italy) (Figure 1). The Park, which covers a surface area of approximately 4950 hectares and is located close to the Pisa city (43°41′ N; 10°19′ E), is characterized by wooded areas with *Quercus robur*, *Quercus ilex*, *Populus alba*, *Fraxinus* spp., *Pinus pinaster*, and *Pinus pinea* as predominant species. Wetlands and agricultural landscapes with meadows and pastures are also present. Fallow deer are largely present in the park with a spring density of 42.8 deer/km^2^ (±8.1 SD), although in the recent past it reached a peak of 99.8 deer/km^2^ [13]. Moreover, several other species occur in the park: farmed horse and cattle, and free-living animals such as wolf (*Canis lupus*), red fox (*Vulpes vulpes*), badger (*Meles meles*), weasel (*Mustela nivalis*), pine marten (*Martes martes*), stone marten (*Martes foina*), and wild boar (*Sus scrofa*). Numerous wild bird species reside in the park, which also serves as a stopover habitat for migratory birds [14].

### 2.2. Samples Collection

A total of 475 ticks were collected from 148 fallow deer (2–6 ticks from each animal) from July to December 2022. The animals were taken by gamekeepers in compliance with the management plan adopted and authorized by the park authority, as previously reported [14]. Technical staff assisted the gamekeepers and collected the ticks immediately after each deer cull. All ticks were gently removed from their hosting animals (2–6 ticks/animal) by forceps or hand and placed into labeled plastic tubes containing 70% ethanol for successive morphological identification performed using standard taxonomic keys [15]. All collected ticks were stored at −20 °C.

Pools were made with 2–6 ticks from the same host. A total of 148 pools, one for each fallow deer, were achieved and submitted to molecular analyses for the pathogens’ detection.

### 2.3. Molecular Analyses

Ticks of each pool were washed with phosphate-buffered saline (PBS), air dried for 10 min on tissue paper and separately sliced into small pieces by a sterile scalpel blade, and then manually homogenized with a sterile micro pestle, resuspended in 200 μL of lysis buffer and 20 μL of proteinase K. After overnight incubation at 56 °C with a continuous gentle shaking, the DNA was extracted using the Zimo research DNA isolation (ZyMO RESEARCH, Quick-DNA™ Kits, cells, tissues, biological fluids, Irvine, CA, USA) following the manufacturer’s protocol. Purified DNA was stored at 4 °C until use.

All DNA samples were submitted to singular PCR assays to detect DNA of bacteria (*A. phagocytophilum*, *Borrelia* sp., *C. burnetii*, *F. tularensis*) and protozoa (*Hepatozoon* sp. and piroplasms). Each PCR reaction was carried out in a 25 µL final volume, containing 12.5 µL EconoTaq PLUS 2× Master Mix (Lucigen Corporation, Middleton, WI, USA), 0.3 µM of each primer, 3 µL of extracted DNA and ultrapure water to reach the final volume. All PCR amplifications were carried out in an automated thermal cycler (SimpliAmp™ Thermal Cycler, Applied Biosystems, Waltham, MA, USA) with the following conditions: 95 °C for 5 min of initial denaturation followed by 40 cycles at 95 °C for 1 min, annealing for 1 min, and 72 °C for 2 min; the reaction was completed by a final step of 10 min at 72 °C. Target genes, primers used, annealing temperatures, and size of the expected amplicons of each protocol are reported in Table 1.

Two nested PCR protocols were used for the detection of *A. phagocytophilum* [10] and *B. burgdorferi* s.l. [11] DNA, respectively. For the detection of piroplasms, a first PCR protocol was used [21]; positive samples were successively subjected to a second PCR assay, amplifying a longer fragment (about 1700 bp) of the 18S rRNA in order to achieve correct species identification with sequencing analyses [22].

For each PCR assay, sterile distilled water was used instead of DNA as a negative control, whereas DNA samples extracted from slides used for indirect immunofluorescent assay (Fuller Laboratories, Torrance, Fullerton, CA; USA) and specific for each investigated pathogen were included as positive controls (Table S1). The obtained PCR products were analyzed by electrophoresis on 1.5% agarose gel at 100 V for 45 min; SharpMass™ 100 Plus Ladder (Euroclone, Milano, Italy) was added as a DNA marker; and gel was stained with ethidium bromide and observed at UV light. In order to confirm the positive results, the amplicons obtained were sequenced by an external laboratory (BMR-Genomics, Padova, Italy). In addition, piroplasms and *Borrelia* amplicons, both obtained with the second PCR protocol, were submitted to sequencing analyses by the same laboratory for a more accurate identification of the pathogen.

The resulting sequences, assembled and corrected through visual inspection of the electropherogram using BioEdit v.7.0.2., were compared with sequences available in GenBank using the BLAST program 2.15.0 (http://www.ncbi.nlm.nih.gov/BLAST, accessed on 12 July 2025).

## 3. Results

All collected ticks were identified as adult female *I. ricinus*.

A total of 102 (68.92%) pools were positive for one or more pathogens. In detail, three (2.02%) for *C. burnetii*, 21 (14.19%) for *Borrelia* sp., 35 (23.64%) for piroplasms, and 87 (58.78%) for *A. phagocytophilum.* All tick pools were negative for *F. tularensis* and *Hepatozoon* sp.

Sixty-seven (45.27%) pools were positive for only one investigated pathogen, whereas in 35 (23.64%) pools two or more pathogens were found: five (3.38%) pools were positive for *A. phagocytophilum*, *Borrelia* sp., and piroplasms; 21 (14.19%) for *A. phagocytophilum* and piroplasms; eight (5.4%) for *A. phagocytophilum* and *Borrelia* sp.; and one (0.67%) for *C. burnetii* and piroplasms.

Sequencing analyses of 28 (18.91%) piroplasm amplicons identified 100% homology with *Theileria* sp. OT3 detected in Spain and Portugal (GenBank n. DQ866840, OL442188); since all sequences from this study were identical, only one sequence was deposited in the GenBank database under accession number PZ028472. All the remaining seven (4.73%) amplicons showed 99.12% homology with *Theileria cervi* (HQ184411); a single sequence was deposited in the GenBank database under accession number PZ044513.

Sequencing of the 21 *Borrelia* amplicons identified six (4.05%) samples as *B. miyamotoi* showing 99.27% homology with the strain found in the French Riviera (MW301924) and 98.47% with two strains identified in *I. ricinus* in the Czech Republic and Netherlands, respectively (KJ847049, CP044783); since all six sequences from this study were identical, only one sequence was deposited in the GenBank database under accession number PZ028451. Eight (5.4%) spirochetal amplicons were identified as *B. lusitaniae* showing 99.84% homology with the strain found in ticks in Slovenia (OZ251982); as all eight sequences were identical, only one was deposited in the GenBank database under accession number PZ028467. The sequences of the remaining seven (4.73%) amplicons had 100% homology with a *Borrelia* sp. found in French Rivier (MW391922) and 99.37% with a *B. theileri* strain detected in Zambia (LC656245); since the seven sequences obtained from this study were identical, a single sequence was deposited in the GenBank database under accession number PZ028469. All results were summarized in Table 2.

## 4. Discussion

The present survey confirmed *I. ricinus* as the tick species most frequently present on the deer population in Central Italy. The geographic study area provides an ideal forest environment for *Ixodes* ticks characterized by leaf litter with sufficient humidity. Moreover, it hosts many wild animal species that can attract *I. ricinus*; in fact, small- to medium-sized mammals are largely present. In addition, given the large blood meal taken by female ticks, the host for these stages is represented by large or intermediate-sized mammals, deer being the most important one [23,24].

Our results, showing a prevalence of 58.78%, confirmed *A. phagocytophilum* as a pathogen widely present in tick populations, particularly in those feeding on deer. Enzootic cycles between ticks and wild animals maintain this pathogen in nature, with *I. ricinus* and wild ruminants most frequently involved [25]. Previous surveys detected prevalences that varied according to geographical area and year of sampling. Values ranging from 4.4% to 63.7% were found in *I. ricinus* sampled in different Italian areas [26,27,28,29,30,31]. In Central Italy, a molecular investigation found a 40% rate for *A. phagocytophilum* in *I. ricinus* collected from roe deer [32], whereas a recent study testing spleens collected from fallow deer living in the San Rossore estate detected the pathogen in 3.08% of the animals [14]. *Anaplasma phagocytophilum* is an obligate intracellular bacterium colonizing granulocyte, mainly neutrophils; it causes diseases mainly in horses, dogs and ruminants, but cases of anaplasmosis have also been observed in humans.

*Borrelia* sp. was detected in 14.19% of the tick pools examined. The low rate found in this study could be related to the fact that the analyzed ticks were removed from fallow deer, which, like other deer species, are considered incompetent hosts [33].

*Borrelia* genus comprises two major groups: the first of them is constituted by *B. burgdorferi* sensu lato (s.l.) complex, the causative agent of Lyme disease, which includes twenty accepted and three proposed genospecies; the second group includes the relapsing fever (RF) *Borrelia* species [10]. Sequencing analyses of our study identified *B. miyamotoi* in six (4.05%) pools. This spirochete, which belongs to the RF group, was reported for the first time in Italy in 2018, specifically in *I. ricinus* specimens collected in the Alpine arch. Previously, it was detected in southern European countries, such as France [34], Portugal [35], and Spain [36]. The low prevalence detected is consistent with findings throughout Europe, where 1.8% of questing *Ixodes* ticks were found on average to be infected [37]. *Borrelia lusitaniae*, which is included in *B. burgdorferi* s.l. complex, has been found in eight (5.4%) pools. *Borrelia lusitaniae* is present in Europe, including Italy; in particular, it has been found in questing ticks, mostly *I. ricinus*, collected in forest and urban and peri-urban areas. In addition, *B. lusitanie* was detected in *Ixodes* ticks feeding on wild ungulates and domestic animals [38]. This *Borrelia* species is maintained in a natural transmission cycle involving tick vectors (*I. ricinus*) and lizards as main reservoirs [39]. *Borrelia miyamotoi* and *B. lusitaniae* are emergent human pathogens which cause symptoms typical of relapsing fever and Lyme disease [39].

Only 3/148 (2.02%) tick pools tested positive for *C. burnetii*. Although the prevalence is low, this finding confirms the role of ticks in the epidemiology of this bacterium, which causes the disease known as Q fever in humans and in domestic and wild animals [18]. However, the role of *I. ricinus* does not appear to be very important in the epidemiology of the pathogen [40], as suggested by analyses of tick populations in Italy, which found 9% of *I. ricinus* positive for *C. burnetii*, compared with 14% of *Dermacentor marginatus* and 25% of *Rhipicephalus turanicus* [41]. Nevertheless, *C. burnetii* was found to be circulating in Central Italy, in agreement with previous studies that found this pathogen in deer and other animals [32,42,43].

All ticks analyzed in this study tested negative for *F. tularensis*. These findings are consistent with those reported in the literature, which described PCR-negative samples collected from different animal species (deer, wild rodents, hares, birds) [42,43,44,45,46]. In recent years, *F. tularensis* has been detected in animals and humans across Europe [47,48,49]; therefore, its monitoring in Italy is advisable in order to assess new epidemiological scenarios and better understand the risk of transmission to humans. In fact, *F. tularensis*, the etiological agent of the zoonosis tularemia, infects several animal species, mainly lagomorphs, and can be transmitted to humans through contact with infected animals, ingestion of contaminated water, as well as tick and mosquito bites [50].

*Hepatozoon* sp. are apicomplexan protozoa affecting many animals, including reptiles, birds and mammals. Usually, the *Hepatozoon* life cycle involves an intermediate (vertebrate animal) and a definitive (arthropod) host; the intermediate host becomes infected by *Hepatozoon* through the ingestion of infected arthropods, mostly ticks [12]. This genus parasitizes several vertebrates, and the risk of spillover among different hosts has recently been emphasized [51]. Ticks of several genera, including *Ixodes*, have been found harboring different *Hepatozoon* species, but all ticks examined in our study tested negative for these pathogens. Our results are quite consistent to those reported in Europe, with an overall low prevalence of about 4% in ticks of different genera (*Dermacentor* 16%, *Ixodes* 5%, and *Rhipicephalus* 1%) [52]. Moreover, *Hepatozoon* sp. appears to be rarely circulating (0.5%) in deer and other Artiodactyla [52].

Regarding piroplasms, *Babesia* sp. was never found in sampled ticks, suggesting a low occurrence of this pathogen in the environment. Previous surveys found low prevalences ranging from 0.1 to 3.8% of *Babesia* sp. (*B. venatorum* and *B. capreoli*) in *I. ricinus* collected in different Italian areas [2,46,53,54,55]. Conversely, *Theileria* parasites were found, alone or in association with bacteria. *Theileria* sp OT3 was the most present piroplasm. This strain has been found in red deer [56] and sheep [57] in Italy. In Europe, little is known about ticks involved in the transmission of *Theileria* to cervids, although genera *Ixodes*, *Hyalomma* and *Rhipicephalus* might be implicated [58]; however, the natural vector and pathogenic significance of this piroplasm have been not yet assessed [59]. *Theileria cervi* has been reported in different cervid species around the world [60] and recently in *D. dama* from the same area [14], although these *Theileria* parasites would not show high specificity to their host species or group, being isolated by equids, too [61].

In our study, 23.64% of the tick pools analyzed were positive for two or more pathogens investigated. The co-presence of more pathogens in ticks has been proven, and it increases the risk of infection for animals and humans. Furthermore, co-infections may increase disease severity, prolong symptom duration, and contribute to atypical or over-lapping clinical presentations, thereby complicating diagnosis and management [62].

From a One Health perspective, the detection of *A. phagocytophilum* and *Borrelia* sp. (including *B. miyamotoi*) in *I. ricinus* pools collected from fallow deer suggests that tick-borne agents of potential public-health relevance circulate in the study area, which is characterized by high wildlife abundance and substantial recreational human use. These pathogens are associated in humans with non-specific febrile syndromes and may therefore remain under-recognized in the absence of a clear history of tick exposure. The molecular detection of *C. burnetii* indicates possible local circulation of the agent; however, human infection is generally linked mainly to inhalation of contaminated aerosols rather than to tick bites. By contrast, the piroplasms detected (*Theileria* sp.) are generally regarded as pathogens of veterinary and ecological importance, and zoonotic transmission has not been reliably documented for the taxa identified. Overall, our findings highlight the value of integrated One Health surveillance to better characterize pathogen circulation at the wildlife–tick–environment interface.

The study has some limitations. First, the number of ticks analyzed was relatively small, and all specimens belonged to I. ricinus. Collecting ticks from a wider range of host animals and using additional methods, such as dragging, could help include other tick species in the analysis. Furthermore, incorporating the analysis of nymphal stages would provide a more comprehensive and accurate picture of the circulation of TBPs.

## 5. Conclusions

The findings of this study highlight the role of ticks in the epidemiology of certain bacteria and protozoa that can cause diseases in animals and humans. In particular, the detection of *B. miyamotoi* and *B. lusitaniae* shows that lesser-known *Borrelia* species are circulating in Italy and represent a risk of infection and disease for humans. Ticks may harbor multiple pathogens simultaneously, increasing the risk of infection in animals and humans, who may develop more severe clinical forms that often are difficult to diagnose. Spending time in forests and other areas with a high density of ticks, such as I. ricinus, is an important risk factor; therefore, preventive measures are pivotal in avoiding TB infections, and monitoring TBPs is an important tool for updating epidemiological scenarios.

## Figures and Tables

**Table 1:** Target genes, primers and annealing temperature for the PCR assays carried out to detect DNA of each pathogen.

Pathogen	Target Gene	Primers Names	Primers Sequences (5′–3′)	Amplicons (bp)	Annealing Temperature (°C)	Ref.
*Anaplasma phagocytophilum*	*16S rRNA* (first PCR)	GE3a GE10r	CACATGCAAGTCGAACGGATTATTC TTCCGTTAAGAAGGATCTAATCTCC	932	55	[16]
	(second PCR)	GE9f GE2	AACGGATTATTCTTTATAGCTTGCT GGCAGTATTAAAAGCAGCTCCAGG	546	55	
*Borrelia* sp.	*16S rRNA* (first PCR)	16S1A 16S1B	CTAACGCTGGCAGTGCGTCTTAAGC AGCGTCAGTCTTGACCCAGAAGTTC	721	63	[17]
	(second PCR)	16S2A 16S2B	AGTCAAACGGGATGTAGCAATAC GGTATTCTTTCTGATATCAACAG	654	56	
*Coxiella burnetii*	*IS1111*	Trans-1 Trans-2	TATGTATCCACCGTAGCCAGT CCCAACAACACCTCCTTATTC	687	64	[18]
*Francisella tularensis*	*TUL4*	TUL4-435	GCTGTATCATCATTTAATAAACTGCTG	400	60.5	[19]
		TUL-863	TTGGGAAGCTTGTATCATGGCACT			
*Hepatozoon* sp.	*18S rRNA*	Hep-F Hep-R	ATACATGAGCAAAATCTCAAC CTTATTATTCCATGCTGCAG	625	57	[20]
Piroplasms	*18S rRNA*	Mic 1 Mic 2	GTCTTGTAATTGGAATGATGG CCAAAGACTTTGATTTCTCTC	560	50	[21]
	*18S rRNA*	Crypto F Crypto R	AACCTGGTTGATCCTGCCAGTAGTCAT GAATGATCCTTCCGCAGGTTCACCTAC	1700	65	[22]

**Table 2:** Co-detection of pathogens in tick pools collected from fallow deer in San Rossore Estate (Tuscany, Italy).

Tick Pool ID	*Anaplasma* *phagocytophilum*	*Borrelia* sp.	*Coxiella burnetii*	*Theileria* sp.
5	+	+ (*B. myiamotoi*)	-	+ (*Theileria* sp. OT3)
8	+	-	-	+ (*T. cervi*)
12	-	-	+	+ (*Theileria* sp. OT3)
21	+	-	-	+ (*T. cervi*)
24	+	+ (*B. lusitaniae*)	-	-
35	+	-	-	+ (*Theileria* sp. OT3)
38	+	+ (*B. lusitaniae*)	-	-
39	+	+ (*B. myiamotoi*)	-	-
43	+	-	-	+ (*Theileria* sp. OT3)
49	+	-	-	+ (*Theileria* sp. OT3)
55	+	-	-	+ (*Theileria* sp. OT3)
68	+	+ (*B. lusitaniae*)	-	+ (*Theileria* sp. OT3)
71	+	-	-	+ (*Theileria* sp. OT3)
74	+	-	-	+ (*Theileria* sp. OT3)
81	+	+ (*B. myiamotoi*)	-	-
86	+	-	-	+ (*Theileria* sp. OT3)
87	+	+ (*B. lusitaniae*)	-	-
93	+	-	-	+ (*Theileria* sp. OT3)
99	+	-	-	+ (*Theileria* sp. OT3)
105	+	+ (*B. myiamotoi*)	-	+ (*Theileria* sp. OT3)
108	+	-	-	+ (*T. cervi*)
112	+	-	-	+ (*Theileria* sp. OT3)
114	+	-	-	+ (*Theileria* sp. OT3)
119	+	+ (*B. lusitaniae*)	-	+ (*Theileria* sp. OT3)
122	+	-	-	+ (*Theileria* sp. OT3)
123	+	+ (*B. lusitaniae*)	-	-
126	+	-	-	+ (*Theileria* sp. OT3)
130	+	-	-	+ (*Theileria* sp. OT3)
131	+	-	-	+ (*Theileria* sp. OT3)
135	+	+ (*B. myiamotoi*)	-	+ (*Theileria* sp. OT3)
139	+	-	-	+ (*T. cervi*)
143	+	-	-	+ (*Theileria* sp. OT3)
144	+	+ (*B. lusitaniae*)	-	-
147	+	-	-	+ (*Theileria* sp. OT3)
148	+	+ (*B. lusitaniae*)	-	-

Legend. ID: identification number; +: positive sample; -: negative sample.

## Data Availability

The original contributions presented in this study are included in the article/Supplementary Material. Further inquiries can be directed to the corresponding author.

## Supplementary Materials

The following supporting information can be downloaded at https://www.mdpi.com/article/10.3390/biology15080596/s1, Table S1. Positive controls included in the PCR assays. Reference [63] is cited in Supplementary Materials.
