# Supplementary material for: Tick-Borne Bacteria and Protozoa in Ixodes ricinus Ticks Collected from Fallow Deer (Dama dama) in a Central Italian Protected Area
Source: Biology (Basel). 2026 Apr 9;15(8):596. doi: 10.3390/biology15080596 (PMC13114137; doi:10.3390/biology15080596)
Supplement: Supplementary file 1 [file biology-15-00596-s001.zip › biology-4227526-supplementary.pdf]

Table S1. Positive controls included in the PCR assays

**Pathogen**	**source**
Anaplasma phagocytophilum	IFAT slide (Fuller Laboratories, Torrance, Fullerton, CA, USA)
Borrelia burgdorferi s.l.	IFAT slide (Fuller Laboratories, Torrance, Fullerton, CA, USA)
Coxiella burnetii	IFAT slide (Fuller Laboratories, Torrance, Fullerton, CA, USA)
Francisella tularensis	IFAT slide (Fuller Laboratories, Torrance, Fullerton, CA, USA)
Hepatozoon sp.	*Hepatozoon canis* positive canine blood sample (Ebani et al, 2015)
Piroplasms	*Babesia caballi/Theileria equi* IFAT slide (Fuller Laboratories, Torrance, Fullerton, CA, USA)

Ebani VV, Nardoni S, Fognani G, Mugnaini L, Bertelloni F, Rocchigiani G, Papini RA, Stefani F, Mancianti F. Molecular detection of vector-borne bacteria and protozoa in healthy hunting dogs from Central Italy. *Asian Pacific J Trop Biomedicine* **2015**, *5*(2), 108-112.
